# ﻿Comparison of caddisfly (Insecta, Trichoptera) assemblages from lake and river habitats of the Huron Mountains of Michigan (USA)

**DOI:** 10.3897/zookeys.1111.70195

**Published:** 2022-07-11

**Authors:** David C. Houghton

**Affiliations:** 1 Department of Biology, Hillsdale College, 33 East College Street, Hillsdale, MI 49242, USA Hillsdale College Hillsdale United States of America

**Keywords:** Functional feeding group, lakes, Michigan, streams, Trichoptera

## Abstract

The caddisfly assemblages of six lakes and 12 1^st^–4^th^ order streams of the Huron Mountains of northern Upper Michigan (USA) were sampled monthly with ultraviolet lights during June-September 2019. A total of 169 species representing 63 genera and 19 families was collected, including five species not found elsewhere in Michigan and two species endemic to the state. Species assemblages between lotic and lentic habitats were distinct from each other, with 11 species indicating lakes and 23 indicating rivers. Despite the taxonomic differences, biomass of functional feeding groups (FFGs) was similar between lakes and rivers, except for higher biomass of predators in the former and higher biomass of filtering collectors in the latter. The FFG biomass of both habitat types was dominated (50–70%) by shredders. Considering the undisturbed condition of the habitats, the caddisfly assemblages and FFG biomass of the Huron Mountains can serve as regional biological monitoring reference conditions.

## ﻿Introduction

Due to the high degradation rates of freshwater habitats, knowledge on the original characteristic assemblages of such habitats is lacking (Ricciardi and Rasmussen 1999; Master et al. 2000; Strayer 2006). Many recent studies have suggested large-scale declines in aquatic insect species (DeWalt et al. 2005; Houghton and Holzenthal 2010; Hawkins and Yuan 2016; Sánchez-Bayo and Wyckhuys 2019; Rhodes 2019; Houghton and DeWalt 2021) or fundamental changes to their community ecology (Baranov et al. 2020; van Klink et al. 2020). Without truly undisturbed reference sites for comparison, however, it is difficult to accurately evaluate current species composition or ecological functioning of freshwater ecosystems. This problem is especially true for lake ecosystems, as research on the biotic assemblages and potential for anthropogenic disturbance of such habitats has lagged far behind that of river habitats (Peck et al. 2020; Fergus et al. 2021). Thus, quantifying assemblages of ecologically important aquatic insect taxa within undisturbed reference sites, especially those of lakes, should be a scientific priority.

The caddisflies (Trichoptera) constitute a particularly important group of organisms for biological monitoring due to their high species richness, ecological diversity, and differing sensitivities to various anthropogenic disturbance (Barbour et al. 1999; Dohet 2002; Houghton 2008; Houghton et al. 2011; Morse et al. 2019a). Although the caddisflies of Michigan are generally well known (Houghton et al. 2018), new species and state records continue to be found in under-collected regions (Houghton 2020). Moreover, nearly all collections of the taxonomically important adult caddisflies in Michigan have consisted of a single sample from a collection site, usually an ultraviolet light trap deployed for a single evening. To accurately capture the characteristic species richness and ecological functioning of Michigan ecosystems, multiple samples would need to be taken from different seasons within a variety of habitats in an undisturbed region.

**Figure 1.**
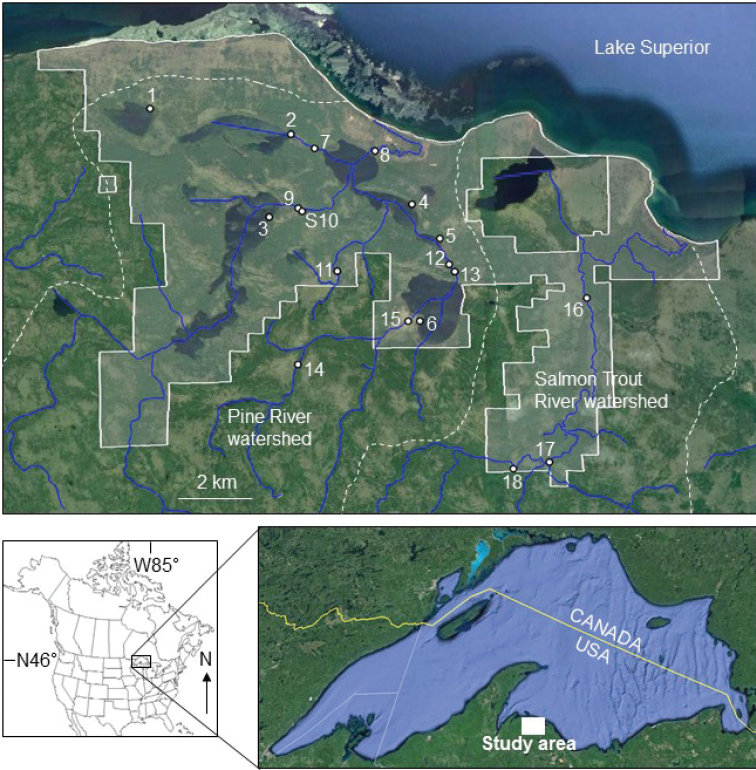
Location of the six lakes and 12 stream sites of the study. Solid white lines denote the approximate borders of the Huron Mountain Club property. Dashed white lines denote the approximate boundaries of the Pine River and Salmon Trout River watersheds. Site numbers correspond to Tables 1, 2. Base maps Google, National Oceanic and Atmospheric Administration, TerraMetrics.

The Huron Mountain Club (HMC) is a ~ 6,000 ha private conservation reserve located in the Huron Mountains of Michigan (Fig. 1). The property is one of the last remaining old-growth mixed hemlock and hardwood forests in the northcentral US (Flader 1983; Yanoviak and McCafferty 1996). Other than some historical and contemporary logging, and a few cabins and small campgrounds, the entire region is undisturbed and has excellent water quality (Woodruff et al. 2010). The HMC contains the middle and lower reaches of the Pine and the Salmon Trout rivers as well as several lakes and smaller tributaries. Due to the undisturbed condition of its habitats, reference conditions have been established for many taxa that occur on the property (www.hmwf.org). When this study began, however, only 21 caddisfly species were known from the HMC (Woods 2011), mostly from Yanoviak and McCafferty’s (1996) study of the benthic communities of the Pine River (Site 8), Mountain Stream (9), and the Salmon Trout River (17) (Fig. 1). The purpose of this study, therefore, was a thorough inventory of the caddisflies of the HMC property to establish reference conditions for species assemblages and ecological functioning within lakes and streams of the region.

## ﻿Materials and methods

**Figure 2.**
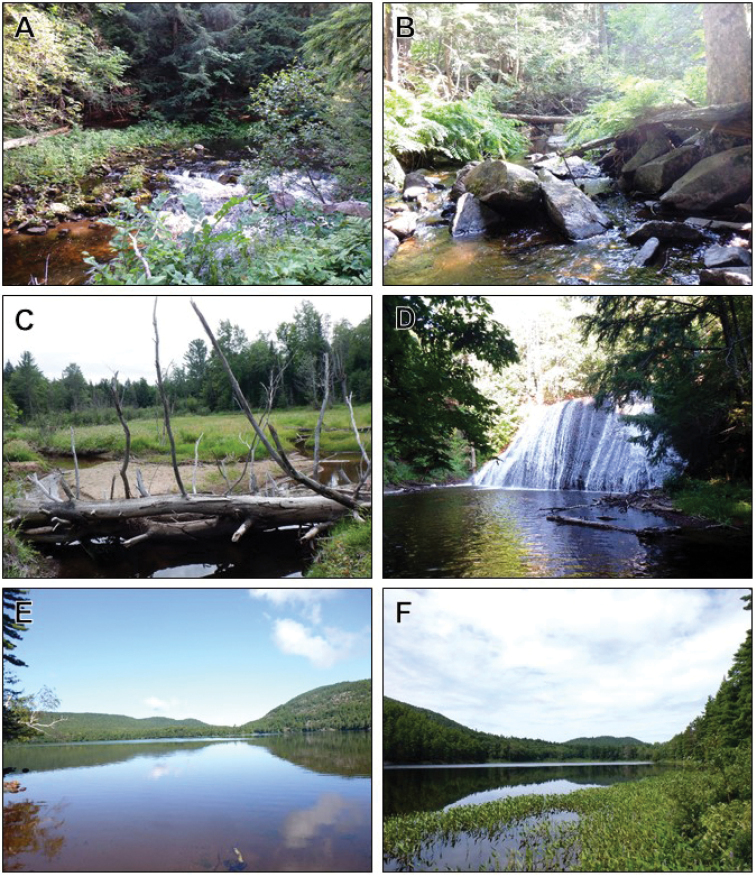
Representative habitats of the Huron Mountains **A** Middle Rapids of the Salmon Trout River (Site 17) **B** River Styx, below the cascade (13) **C** multiple braided channels of the North Fork of Elm Greek (14) **D** pool below the falls of Mountain Stream (10) **E** Mountain Lake (3) **F** Third Pine Lake (5). Site numbers correspond to Fig. 1 and Tables 1, 2. Photographs taken August 2019.

Six lakes and 12 stream sites were chosen for caddisfly sampling (Fig. 1, Tables 1, 2). Sites were chosen to reflect a variety of habitats (Fig. 2) that also had reasonable road access. Several rivers were sampled at more than one location. One site was just outside the HMC property. There were no dams or human settlements within the watersheds of any of the study sites.

**Table 1.** The 18 sites sampled during this study with the total number of caddisfly species caught at each site. Site numbers correspond to Fig. 1 and Table 2. All sites were sampled once during June, July, August, and September 2019. Mean species richness was the same in rivers as in lakes based on a non-parametric Mann-Whitney *U*-test between the habitat types (*P* = 0.065).

Site	Location	Latitude / Longitude	Elevation (m)	species
1	Howe Lake, northeast boathouse	46.8932°, -87.9436°	211	41
2	Rush Lake, east boathouse	46.8869°, -87.8967°	195	55
3	Mountain Lake, east boathouse	46.8681°, -87.9043°	258	48
4	Second Pine Lake, east boathouse	46.8705°, -87.8567°	185	42
5	Third Pine Lake, eastern picnic area	46.8626°, -87.8475°	186	44
6	Ives Lake, west side, at Stonehouse,	46.8439°, -87.8547°	232	53
**Mean of lakes 47 (±3.4)**				
7	Rush Creek, Mountain Lake Road	46.8836°, -87.8889°	187	70
8	Pine River, main entrance road	46.8828°, -87.8687°	184	71
9	Mountain Stream, at bridge	46.8699°, -87.8946°	227	48
10	Mountain Stream, below waterfall	46.8692°, -87.8933°	216	41
11	Fisher Creek, Loop Road	46.8555°, -87.8819°	250	44
12	River Styx, entrance foot bridge	46.8567°, -87.8446°	187	65
13	River Styx, base of cascade	46.8550°, -87.8428°	205	55
14	North Fork, Elm Creek, Loop Road	46.8377°, -87.8975°	248	64
15	Elm Creek, near Stonehouse	46.8439°, -87.8586°	233	52
16	Salmon Trout River, entrance bridge	46.8485°, -87.7989°	192	57
17	Salmon Trout River, Middle Falls	46.8100°, -87.8245°	223	50
18	Salmon Trout River, Lower Dam	46.8114°, -87.8125°	218	79
**Mean of rivers 58** (±**2.4)**				

**Table 2.** Physicochemical data for the 18 sites of this study. Site numbers correspond to Table 1 and Fig. 1. See Materials and methods for further explanation of how data were obtained.

	Lake sites						River sites											
Parameter	1	2	3	4	5	6	7	8	9	10	11	12	13	14	15	16	17	18
pH	8.4	8.4	8.4	8.0	8.2	8.4	8.5	8.0	8.3	8.3	8.6	8.4	8.4	8.3	8.1	8.0	8.1	8.1
DO (mg/L)	7.4	8.1	7.9	7.2	7.4	7.9	8.9	8.2	8.4	8.4	8.6	7.2	7.6	9.0	7.2	8.5	9.1	9.1
K (µC/cm^2^)	40	70	100	80	80	60	60	80	100	100	90	60	60	90	100	110	120	120
Stream temperature (°C)			N/A				14.8	17.2	16.1	16.1	14.5	15.2	15.2	14.5	14.8	16.9	15.7	15.7
Width (m)			N/A			2	15	8	8	3	6	3	3	6	11	7	7	
Area (ha)	68	125	332	71	23	191						N/A						
Shoreline (km)	3.8	8.7	16.3	4.9	2.4	6.1						N/A						
Maximum depth (m)	15	90	20	14	5	34						N/A						
Mean depth (m)	5	22	6	3	1.5	9.4						N/A						
Velocity (m/s)			N/A				0.7	0.4	3.2	0.7	0.3	0.2	0.6	0.6	0.2	0.3	2.5	2.3
Sinuosity			N/A				1.16	1.84	1.15	1.15	1.58	1.24	1.18	1.75	1.83	1.47	1.24	1.25
Percent intact habitat	95	94	94	95	95	95	93	97	94	94	97	98	98	93	78	96	95	95
Percent exotic plants	0.1	0.1	4.9	0.0	0.0	5.4	1.9	8.2	4.8	3.0	3.0	4.2	4.2	3.5	4.4	4.3	6.8	6.8
Percent base flow	61	61	61	62	62	62	62	61	61	61	61	62	62	61	62	62	62	62
Distance to bedrock (cm)	89	89	130	89	89	138	89	89	130	130	130	138	138	130	130	140	130	130
CTI	587	587	851	653	653	932	460	645	387	387	395	470	470	419	799	448	355	355
Distance to H_2_O table (cm)	178	178	181	157	157	142	157	157	181	181	181	142	142	182	182	152	182	182
Percent impervious surface	0.04	0.04	0.03	0.03	0.03	0.26	0.08	0.38	0.04	0.04	0.02	0.02	0.02	0.14	0.17	0.05	0.03	0.03
Percent soil organic matter	1.5	1.5	0.5	0.8	0.8	0.5	3.5	3.5	0.5	0.5	0.5	3.0	3.0	0.5	0.5	2.7	0.5	0.5
Soil permeability (cm/h)	12	12	32	12	12	23	12	12	32	32	32	23	23	32	32	26	32	32
Roads (km/km^2^)	0.7	0.7	0.4	0.6	0.6	0.6	1.5	3.1	0.7	0.7	0.8	0.6	0.6	1.1	1.6	0.9	0.9	0.9

In total, 23 environmental variables were measured at each site or obtained from other sources. Some variables applied only to streams, others only to lakes, and others to both habitat types (Table 2). Latitude, longitude, and elevation were determined using Google Earth Pro (GE), as was width at each stream site. Stream sinuosity was determined in GE by tracing the stream for ~ 2 km upstream of each sampling site and dividing by the straight line distance between the beginning and end of the trace (Gordon 2004). Some smaller tributaries necessitated traces < 2 km. Physicochemical stream variables were measured during a 4-day period during August 2019. This period was chosen to maximize leaf abundance on trees while minimizing stream flow variation. No rain events occurred during the 4-day period. Twelve measurements of specific conductance (ECTestr Low, www.eutechinst.com), pH (AccuMetAP61, www.fishersci.com), flow velocity (Flowatch, www.jdc.ch), and dissolved oxygen (YSI-55, OH,www.ysi.com) were taken near each sampling site within a 10-min period and the mean value was recorded. Measurements were taken for all sites within 2 h. This procedure was repeated over the subsequent 3 days, and a global mean was determined for each variable. Total area, total shoreline perimeter, maximum depth, and mean depth were determined for each lake from an internal bathymetry report of the property (www.hmwf.org).

Several other site variables were determined using the USEPA StreamCat database (https://watersgeo.epa.gov/watershedreport), accessed November 2020 (Hill et al. 2016). These variables included: percentage of base flow relative to total flow, distance from stream bottom to bedrock, distance from stream bottom to water table, percentage of organic matter by volume in the soil, soil permeability, mean composite topographic index (CTI), percentage of impervious surface, density of roads, percentage of plant cover not native to the region, and overall percentage of undisturbed (forest or wetland) land cover. All of these variables were at the local (HUC-12) catchment level. In addition, mean summer stream temperature was determined for each specific site, also from the StreamCat database.

Sampling for caddisfly adults occurred during 2019. An ultraviolet blacklight sample was collected from each site in June, July, August, and September, for a total of four samples from each site. Each sample consisted of a 10-watt portable ultraviolet LED light placed over a white pan filled with 80% ethanol (Zemel and Houghton 2017). Lights were placed ~ 1 m from each site, turned on at dusk, and collected ~ 1 h after dusk (Wright et al. 2013). Samples were collected only if the peak daytime temperature was > 25° C, dusk temperature was > 18° C, and there was no noticeable wind or precipitation at dusk (Houghton 2004). Each set of monthly samples was taken within four days of each other. Since aquatic insects collected within 40 m of a habitat accurately reflect the assemblage of that habitat (Sode and Wiberg-Larson 1993; Peterson et al. 1999; Sommerhäuser et al. 1999; Brakel et al. 2015), dispersals of adults between sites, while certainly possible, were considered unimportant.

Specimens were identified using Houghton’s (2012) treatment of the Minnesota caddisflies or with more specific taxonomic treatments as needed. Specimens were coded with their affinity for one of six different functional feeding groups (FFGs) based on Morse et al. (2019b) and some unpublished gut content analyses: algal piercers, filtering collectors, gathering collectors, predators, scrapers, and shredders. Codes consisted of ‘0’ for no affinity for a FFG, ‘1’ low affinity, ‘2’ moderate affinity, ‘3’ high affinity, and ‘4’ near exclusive affinity (Chevenet et al. 1994) (Table 3). These codes were converted to proportions: 0 = 0.0, 1 = 0.25, 2 = 0.50, 3 = 0.75, and 4 = 1.0, to multiply by the determined biomass for each genus (Beauchard et al. 2017). This approach more accurately reflected the feeding plasticity of aquatic insects than pure categorization (Dolédec et al. 2000; Gayraud et al. 2003; Tomanova et al. 2007).

**Table 3.** The 169 caddisfly species collected during this study, showing total number of localities (#locs) and total number of specimens (#spcs), and mean ash-free dry mass (AFDM) (mg) from lakes and rivers. Species are organized alphabetically by family and genus. Asterisks denote significant affinity with lakes or rivers based on indicator species analysis. Functional feeding groups (FFGs) as follows: FC = filtering collector, GC = gathering collector, Pi = algal piercer, Pr = predator, Sc = scraper, Sh = shredder.

	FFG affinity coding									
Taxon	FC	GC	Pi	Pr	Sc	Sh	# locs	#spcs	AFDM (lakes)	AFDM (rivers)
BRACHYCENTRIDAE (2)										
*Brachycentrusamericanus* (Banks, 1899)	3	0	0	0	0	1	4	29	0.000	1.801
*Micrasemawataga* Ross, 1938	1	1	0	0	0	2	6	103	0.016	0.801
DIPSEUDOPSIDAE (1)										
*Phylocentropusplacidus* (Banks, 1905)	4	0	0	0	0	0	11	136	2.579	3.450
GLOSSOSOMATIDAE (3)										
*Glossosomaintermedium* Klapálek, 1892	0	0	0	0	4	0	9	113	0.047	2.654*
*G.nigrior* Banks, 1911	0	0	0	0	4	0	8	549	0.000	13.009*
*Protoptilatenebrosa* (Walker, 1852)	0	0	0	0	4	0	1	4	0.000	0.010
GOERIDAE (1)										
*Goerastylata* Ross, 1938	0	0	0	0	4	0	3	109	0.000	4.495*
HELICOPSYCHIDAE (1)										
*Helicopsycheboreali*s (Hagen, 1861)	0	0	0	0	4	0	12	773	12.629	8.041
HYDROPSYCHIDAE (15)										
*Arctopsycheladogensis* (Kolenati, 1859)	3	0	0	0	0	1	2	101	0.000	1.608
*Cheumatopsycheanalis* (Banks, 1908)	4	0	0	0	0	0	11	76	0.115	2.133*
*C.campyla* Ross 1938	4	0	0	0	0	0	11	484	3.401	12.249*
*C.gracilis* (Banks, 1899)	4	0	0	0	0	0	8	263	0.058	7.551*
*C.oxa* Ross, 1938	4	0	0	0	0	0	3	6	0.040	0.102
*Hydropsychealhedra* (Ross, 1939)	4	0	0	0	0	0	2	39	0.000	1.273
*H.betteni* Ross, 1938	4	0	0	0	0	0	11	174	1.370	9.249*
*H.morosa* (Hagen, 1861)	4	0	0	0	0	0	10	357	0.196	11.557*
*H.slossonae* (Banks, 1905)	4	0	0	0	0	0	5	87	0.000	2.840*
*H.sparna* (Ross, 1938)	4	0	0	0	0	0	13	722	0.678	26.843*
*H.vexa* (Ross, 1938)	4	0	0	0	0	0	1	3	0.000	0.098
*H.walkeri* (Betten and Mosely, 1940)	4	0	0	0	0	0	4	22	0.000	0.719
*Macrostemumzebratum* (Hagen, 1861)	4	0	0	0	0	0	1	2	0.000	0.295
*Parapsycheapicalis* (Banks, 1908)	3	0	0	0	0	1	2	2	0.000	0.079
*Potamyiaflava* (Hagen, 1861)	4	0	0	0	0	0	2	2	0.079	0.039
HYDROPTILIDAE (37)										
*Agrayleamultipunctata* Curtis, 1834	0	2	2	0	0	0	9	24	0.025	0.047
*Hydroptilaalbicornis* Hagen, 1861	0	0	3	0	1	0	1	1	0.001	0.000
*H.amoena* Ross, 1938	0	0	3	0	1	0	7	17	0.003	0.022
*H.ampoda* Ross, 1941	0	0	3	0	1	0	4	17	0.003	0.022
*H.antennopedia* Sykora and Harris, 1994	0	0	3	0	1	0	1	1	0.000	0.001
*H.consimilis* Morton, 1905	0	0	3	0	1	0	4	10	0.000	0.014
*H.hamata* Morton, 1905	0	0	3	0	1	0	3	30	0.003	0.040
*H.fiskei* Blickle, 1963	0	0	3	0	1	0	4	8	0.002	0.009
*H.jackmanni* Blickle, 1963	0	0	3	0	1	0	6	103	0.003	0.141
*H.novicola* Blickle & Morse, 1954	0	0	3	0	1	0	1	1	0.000	0.001
*H.salmo* Ross, 1941	0	0	3	0	1	0	1	1	0.000	0.001
*H.tortosa* Ross, 1938	0	0	3	0	1	0	1	1	0.001	0.000
*H.valhalla* Denning, 1947	0	0	3	0	1	0	5	8	0.000	0.011
*H.waubesiana* Betten, 1934	0	0	3	0	1	0	1	1	0.003	0.000
*H.wyomia* Denning, 1948	0	0	3	0	1	0	1	2	0.000	0.003
*H.xera* Ross, 1938	0	0	3	0	1	0	7	41	0.000	0.057
*Ithytrichiaclavata* Morton, 1905	0	0	1	0	3	0	4	8	0.000	0.011
*Leucotrichiapictipes* (Banks, 1911)	0	0	2	0	2	0	1	1	0.000	0.001
*Mayatrichiaayama* Mosely, 1905	0	0	1	0	3	0	2	2	0.003	0.001
*Neotrichiahalia* Denning, 1948	0	0	0	0	4	0	3	9	0.002	0.008
*N.okopa* Ross, 1939	0	0	0	0	4	0	1	1	0.000	0.001
*Ochrotrichiatarsalis* (Hagen, 1861)	0	1	3	0	0	0	1	1	0.000	0.001
*Orthotrichiaaegerfasciella* (Chambers, 1873)	0	0	4	0	0	0	3	21	0.007	0.014
*O.balduffi* Kingsolver & Ross, 1961	0	0	4	0	0	0	3	7	0.000	0.007
*O.cristata* Morton, 1905	0	0	4	0	0	0	4	23	0.040	0.002
*O.curta* Kingsolver & Ross, 1961	0	0	4	0	0	0	4	19	0.015	0.011
*Oxyethiraaraya* Ross, 1941	0	1	3	0	0	0	1	1	0.000	0.001
*O.coercens* Morton, 1905	0	1	3	0	0	0	4	39	0.006	0.034
*O.forcipata* Mosely, 1934	0	1	3	0	0	0	5	7	0.000	0.007
*O.michiganensis* Mosely, 1934	0	1	3	0	0	0	8	48	0.000	0.046
*O.obtatus* Denning, 1947	0	1	3	0	0	0	2	3	0.004	0.001
*O.rivicola* Blickle & Morse, 1954	0	1	3	0	0	0	7	21	0.000	0.020
*O.sida* Blickle & Morse, 1954	0	1	3	0	0	0	2	8	0.005	0.006
*O.verna* Ross, 1938	0	1	3	0	0	0	1	1	0.000	0.001
*O.zeronia* Ross, 1941	0	1	3	0	0	0	1	1	0.000	0.001
*Stactobielladelira* (Ross, 1938)	0	1	3	0	0	0	1	1	0.000	0.001
*S.palmata* (Ross, 1938)	0	1	3	0	0	0	1	3	0.003	0.000
LEPIDOSTOMATIDAE (6)										
*Lepidostomabryanti* (Banks, 1908)	0	1	0	0	0	3	15	536	1.055	19.662*
*L.griseum* (Banks, 1911)	0	1	0	0	0	3	2	9	0.000	0.339
*L.sackeni* (Banks, 1936)	0	1	0	0	0	3	2	2	0.000	0.078
*L.togatum* (Hagen, 1861)	0	1	0	0	0	3	16	1835	21.261	61.087
*L.unicolor* (Banks, 1911)	0	1	0	0	0	3	4	22	0.000	0.860
*L.vernale* (Banks, 1897)	0	1	0	0	0	3	2	3	0.000	0.117
LEPTOCERIDAE (34)										
*Ceracleaalagma* (Ross, 1938)	0	2	0	1	0	1	5	37	4.169*	0.058
*C.ancylus* (Vorhies, 1909)	0	2	0	1	0	1	6	4	0.463	0.000
*C.arielles* (Denning, 1942)	0	2	0	1	0	1	3	420	0.000	11.131*
*C.cancellata* (Betten, 1942)	0	2	0	1	0	1	6	31	3.127	0.232
*C.excisa* (Morton, 1904)	0	2	0	1	0	1	1	1	0.114	0.000
*C.flava* (Ross, 1904)	0	2	0	1	0	1	1	1	0.000	0.057
*C.maculata* (Banks, 1899)	0	2	0	1	0	1	1	16	1.817	0.000
*C.resurgens* (Walker, 1852)	0	2	0	1	0	1	12	266	2.731	14.428
*C.tarsipunctata* (Vorhies, 1909)	0	2	0	1	0	1	13	205	17.491*	2.896
*C.transversa* (Hagen, 1861)	0	2	0	1	0	1	14	210	13.318	5.5009
*Leptocerusamericanus* (Banks, 1899)	0	1	0	0	0	3	4	5	0.156	0.020
*Mystacidesinterjecta* (Banks, 1914)	0	3	0	0	0	1	4	72	3.745*	0.053
*M.sepulchralis* (Walker, 1852)	0	3	0	0	0	1	9	88	3.638	0.535
*Nectopsychealbida* (Walker, 1852)	0	1	0	0	0	3	2	24	2.277	0.049
*N.exquisita* (Walker, 1852)	0	1	0	0	0	3	4	25	2.474	0.000
*N.pavida* (Hagen, 1861)	0	1	0	0	0	3	7	167	1.568	2.063
*Oecetisavara* (Banks, 1895)	0	1	0	2	0	1	7	315	0.418	10.769*
*O.cinerascens* (Hagen, 1861)	0	1	0	2	0	1	12	284	20.124*	0.641
*O.immobilis* (Hagen, 1861)	0	1	0	2	0	1	2	2	0.151	0.000
*O.inconspicua* (Walker, 1852)	0	1	0	2	0	1	18	3370	221.438*	16.280
*O.nocturna* Ross, 1966	0	1	0	2	0	1	1	2	0.151	0.000
*O.osteni* Milne, 1934	0	1	0	2	0	1	10	169	10.136	0.798
*O.persimilis* (Banks, 1907)	0	1	0	2	0	1	10	205	3.332	5.450
*O.sordida* (Blahnik and Holzenthal, 2014)	0	1	0	2	0	1	5	84	0.377	2.977
*Setodesincertus* (Walker, 1852)	0	3	0	1	0	0	2	4	0.064	0.032
*S.truncatus* Houghton, 2021	0	3	0	1	0	0	2	4	0.000	0.096
*Triaenodesabus* Milne, 1935	0	1	0	0	0	3	2	2	0.099	0.0460
*T.baris* Ross, 1938	0	1	0	0	0	3	3	4	0.199	0.099
*T.dipsius* Ross, 1938	0	1	0	0	0	3	5	12	0.694	0.248
*T.ignitus* (Walker, 1852)	0	1	0	0	0	3	4	34	0.000	1.684
*T.injustus* (Hagen, 1861)	0	1	0	0	0	3	10	339	29.827*	1.883
*T.marginatus* Sibley, 1926	0	1	0	0	0	3	5	77	1.883	2.874
*T.perna* Ross, 1938	0	1	0	0	0	3	1	1	0.099	0.000
*T.tardus* Milne, 1934	0	1	0	0	0	3	8	12	0.396	0.396
LIMNEPHILIDAE (29)										
*Anaboliabimaculata* (Walker, 1852)	0	1	0	0	0	3	7	8	1.206	1.005
*A.consocia* (Walker, 1852)	0	1	0	0	0	3	5	5	0.308	0.616
*Asynarchusmontanus* (Banks, 1907)	0	1	0	0	0	3	2	8	0.000	1.608
*A.rossi* Leonard & Leonard, 1949	0	1	0	0	0	3	1	5	0.000	1.005
*Hesperophylaxdesignatus* (Walker, 1852)	0	1	0	0	0	3	2	2	0.000	0.662
*Hydatophylaxargus* (Harris, 1869)	0	1	0	0	0	3	11	59	2.174	30.974*
*Ironoquialyrata* (Ross, 1938)	0	0	0	0	0	4	2	2	0.000	0.266
*Lenarchuscrassus* (Banks, 1920)	0	3	0	0	0	1	1	1	0.000	0.133
*Limnephilusargenteus* Banks, 1914	0	1	0	0	0	3	1	1	0.000	0.133
*L.indivisus* Walker, 1852	0	1	0	0	0	3	3	8	0.000	1.530
*L.infernalis* (Banks, 1914)	0	1	0	0	0	3	7	34	12.239*	0.382
*L.femoralis* Kirby, 1837	0	1	0	0	0	3	1	1	0.000	0.133
*L.moestus* Banks, 1908	0	1	0	0	0	3	15	89	3.356	9.809
*L.ornatus* Banks, 1907	0	1	0	0	0	3	10	36	1.549	3.872
*L.rhombicus* (L., 1758)	0	1	0	0	0	3	2	5	0.000	0.645
*L.sericeus* (Say, 1824)	0	1	0	0	0	3	9	28	2.323	2.452
*L.submonilifer* Walker, 1852	0	1	0	0	0	3	8	18	0.774	1.936
*L.thorus* Ross, 1938	0	1	0	0	0	3	1	1	0.000	0.129
*Nemotauliushostilis* (Hagen, 1873)	0	0	0	0	0	4	1	1	0.000	0.460
*Onocosmoecusunicolor* (Banks, 1897)	0	0	0	0	0	4	10	290	1.182	56.503*
*Platycentropusradiatus* (Say, 1824)	0	0	0	0	0	4	14	55	11.258	12.582
*Pseudostenophylaxsparsus* (Banks, 1908)	0	1	0	0	0	3	9	16	0.797	1.728
*Pycnopsycheaglona* Ross 1941	0	0	0	0	1	3	4	99	2.93	16.677
*P.antica* (Walker, 1852)	0	0	0	0	1	3	12	267	1.181	51.975*
*P.circularis* (Provancher, 1877)	0	0	0	0	1	3	12	126	1.466	22.358*
*P.guttifera* (Walker, 1852)	0	0	0	0	1	3	17	1088	85.767	156.507
*P.lepida* (Hagen, 1861)	0	0	0	0	1	3	10	134	2.932	23.091
*P.limbata* (MacLachlan, 1871)	0	0	0	0	1	3	6	12	0.367	2.016
*P.subfasciata* (Say, 1828)	0	0	0	0	1	3	10	218	74.039*	2.932
MOLANNIDAE (4)										
*Molannablenda* Sibley, 1926	0	1	0	1	2	0	8	69	0.000	3.943*
*M.flavicornis* Banks, 1914	0	1	0	1	2	0	2	4	0.358	0.056
*M.tryphena* Betten, 1934	0	1	0	1	2	0	7	75	0.000	4.472*
*M.uniophila* Vorhies, 1909	0	1	0	1	2	0	13	664	59.505*	9.838
ODONTOCERIDAE (1)										
*Psilotretaindecisa* (Walker, 1852)	0	1	0	0	3	0	2	103	0.000	6.193
PHILOPOTAMIDAE (4)										
*Chimarraferia* (Ross, 1941)	4	0	0	0	0	0	3	5	0.000	0.148
*C.obscura* (Walker, 1852)	4	0	0	0	0	0	7	51	0.236	1.387
*Dolophilodesdistinctus* (Walker, 1852)	4	0	0	0	0	0	11	374	0.131	12.221*
*Wormaldiamoesta* (Banks, 1914)	4	0	0	0	0	0	2	2	0.000	0.066
PHRYGANEIDAE (8)										
*Agrypniaimproba* (Hagen, 1873)	0	0	0	0	0	4	6	22	0.510	5.353
*A.vestita* (Walker, 1852)	0	0	0	0	0	4	4	4	1.529	0.255
*Banksiolacrotchi* Banks, 1844	0	0	0	1	0	3	18	370	22.162	31.187
*B.dossuaria* (Say, 1828)	0	0	0	1	0	3	3	12	0.735	1.103
*Hagenellacanadensis* (Banks, 1907)	0	0	0	1	0	3	2	2	0.000	0.510
*Phryganeacinerea* Walker, 1852	0	0	0	1	0	3	14	55	25.101	18.826
*Ptilostomisocellifera* (Walker, 1852)	0	0	0	1	0	3	13	66	16.839	31.272
*P.semifasciata* (Say, 1828)	0	0	0	1	0	3	17	85	40.896	30.672
POLYCENTROPODIDAE (15)										
*Cernotinapallida* (Banks, 1904)	1	0	0	3	0	0	3	38	0.668*	0.000
*Holocentropusflavus* Banks, 1908	1	0	0	3	0	0	4	11	0.000	0.383
*H.interruptus* Banks, 1914	1	0	0	3	0	0	5	6	0.170	0.170
*Neureclipsiscrepuscularis* (Walker, 1852)	2	0	0	1	0	1	9	116	0.824	1.721
*Nyctiophylaxaffinis* (Banks, 1897)	1	0	0	2	0	1	6	248	1.627	0.734
*N.moestus* Banks, 1911	1	0	0	2	0	1	9	57	0.631	1.678
*Plectrocnemiaalbipuncta* Banks, 1930	1	0	0	3	0	0	8	50	0.083	0.649
*P.cinerea* (Hagen, 1861)	1	0	0	3	0	0	11	103	2.016*	0.400
*P.clinei* Milne, 1936	1	0	0	3	0	0	3	5	0.000	0.069
*P.icula* (Ross, 1941)	1	0	0	3	0	0	4	33	0.000	0.456
*P.remota* (Banks, 1911)	1	0	0	3	0	0	6	8	0.000	0.278
*P.sabulosa* (Leonard & Leonard, 1949)	1	0	0	3	0	0	3	11	0.000	0.383
*Polycentropuscentralis* Banks, 1914	1	0	0	3	0	0	1	5	0.000	0.069
*P.confusus* Hagen, 1861	1	0	0	3	0	0	16	336	0.387	4.446
*P.pentus* Ross, 1941	1	0	0	3	0	0	6	43	0.000	1.496
*P.timesis* (Denning, 1948)	1	0	0	3	0	0	1	1	0.000	0.035
PSYCHOMYIIDAE (2)										
*Lypediversa* (Banks, 1914)	0	2	0	0	2	0	15	420	0.096	1.298*
*Psychomyiaflavida* Hagen, 1861	0	3	0	0	1	0	15	178	0.081	0.516
RHYACOPHILIDAE (2)										
*Rhyacophilabrunnea* Banks, 1911	0	1	0	3	0	0	1	4	0.000	0.151
*R.fuscula* (Walker, 1852)	0	1	0	3	0	0	6	305	0.234	35.506*
SERICOSTOMATIDAE (1)										
*Agarodesdistinctus* (Ulmer, 1905)	0	2	0	0	0	2	9	60	4.640	1.657
THREMMATIDAE (2)										
*Neophylaxconcinnus* McLachlan, 1871	0	0	0	0	0	4	4	14	0.055	0.356
*N.oligius* Ross, 1938	0	0	0	0	0	4	9	271	0.000	7.422*

Ash-free dry mass (AFDM) values for each species were taken from Houghton and Lardner’s (2020) determination of 63 common caddisflies of the north-central US. Species without a determined value were assigned the value of a congener of similar size. While this approach did not reflect differences in body size due to differences in sexual dimorphism, specific habitat, larval food quality, or emergence timing, among other differences (Svensson 1975; Wagner 2002; Wagner 2005), it still allowed for a more precise determination of FFG differences between sites than simply counting specimens and treating them as ecologically equivalent, while also preserving the vast majority as vouchers. All specimens have been deposited in the Hillsdale College Insect Collection (HCIC).

To delineate differences between caddisfly assemblages of lake and river habitats, specimens were examined with a non-metric multidimensional scaling (NMDS) ordination using the program PC-ORD v.7 for Windows (Peck 2016). The data matrix consisted of log_10_ (x + 1) transformed specimen counts per site for each species for each of the monthly samples. The mean of these four values was then determined for each site for each species. All species were weighted equally. The NMDS ordination was conducted using the default program settings, 250 randomized runs, and a Bray-Curtis distance measure. A Monte Carlo test was conducted on each determined axis to assess its difference from a random ordination structure (Dexter et al. 2018). Since several important stream variables (e.g., width) are not appropriate for analyzing lakes, and others (e.g., flow velocity) may lead to artificial continua from lakes to slow-moving rivers, no secondary matrix of environmental variables was correlated with the primary matrix. Differences in mean biomass for each FFG between lakes and streams were determined using non-parametric Mann-Whitney *U*-tests.

Species important for indicating lake or river habitats were determined with Dufrêne and Legendre’s (1997) indicator species technique, also using PC-ORD. This method determines a species’ indicator value based on a combination of the percentage of habitats that contain a particular species, and the average abundance of that species within each habitat type divided by the average abundance of that species in all habitat types. Thus, in order to be a significant indicator of either lakes or rivers, a species needed to be common and abundant in the respective habitat type only.

## ﻿Results

A total of 21,235 specimens were collected and identified, representing 169 species within 63 genera and 19 families (Table 3). Hydroptilidae (37), Leptoceridae (34), and Limnephilidae (29) were the most species-rich families. *Hydroptila* (15), *Ceraclea* (10), and *Limnephilus* (10) were the most species-rich genera.

*Pycnopsycheguttifera* (Walker) (Limhephilidae) (2392 mg) had the highest overall AFDM, followed by *Oecetisinconspicua* (Walker) (Leptoceridae) (1524), *Lepidostomatogatum* (Hagen) (Lepidostomatidae) (861), and *Onoconsmoecusunicolor* (Banks) (Limnephilidae) (685) (Table 3). Over half of the AFDM of the entire assemblage was represented collectively by the species of *Pycnopsyche* (28%), *Oecetis* (13%), *Lepidostoma* (7%), and *Ptilostomis* (7%). *Banksiolacrotchi* Banks (Phryganeidae) and *Oecetisinconspicua* were found at all 18 sites; *Ptilostomissemifasciata* (Say) (Phryganeidae) and *Pycnopsycheguttifera* were found at 17 sites. Thirty-one species were found at only a single site.

**Figure 3.**
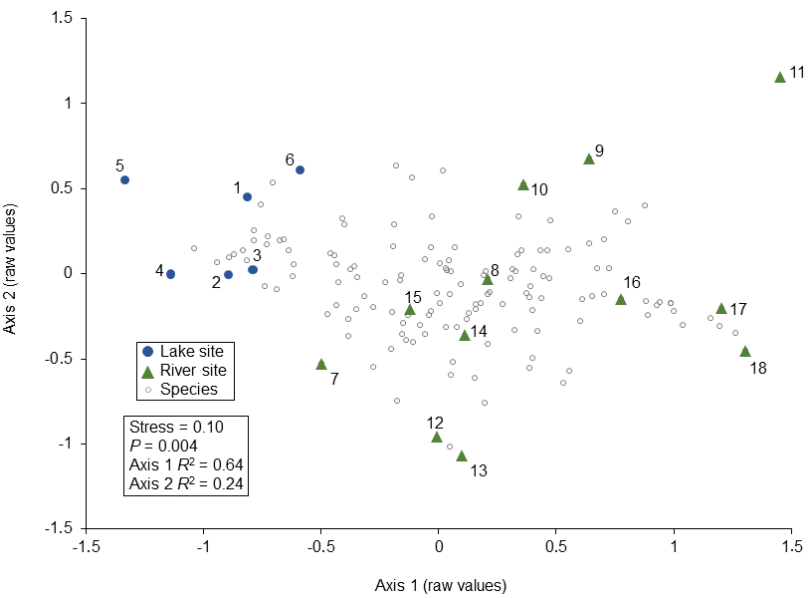
NMDS ordination of the 18 sampling sites based on caddisfly log_10_ specimen abundance per species per site, and reflecting the combined four samples for each site. *P*-values from a Monte Carlo test of non-random ordination structure. Site numbers correspond to Fig. 1 and Tables 1, 2. Species labels omitted for clarity.

**Figure 4.**
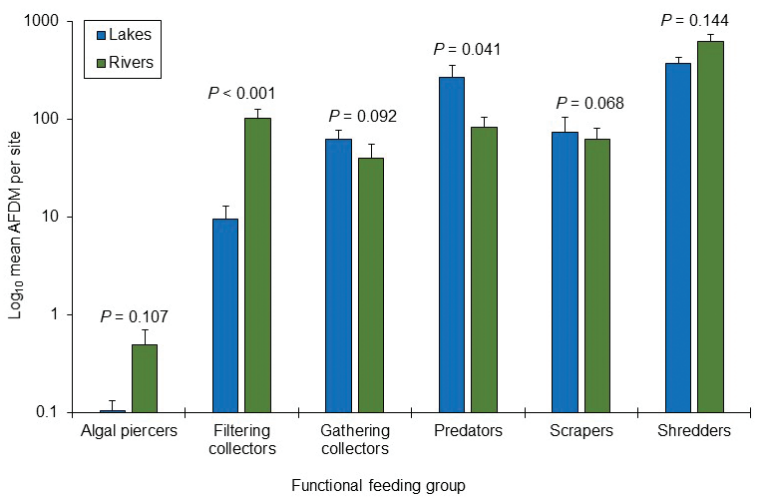
Log_10_ mean (+SE) total AFDM for caddisfly FFGs between lakes and rivers of the Huron Mountains. *P*-values based on nonparametric Mann-Whitney *U*-tests of the mean biomass for each FFG between lake and river habitats. *N* = six for lakes and 12 for rivers.

An NMDS ordination of species assemblages for all sampling sites produced a two-dimensional solution explaining almost 90% of the variation in the data set (Fig. 3). Lake and river sampling sites were distinct from each other with no overlap. Mean species richness was similar in river (58) and lake (47) habitats (Table 1). Mean biomass was not different between lake and river sites for any FFG, except for higher filtering collectors in rivers and higher predators in lakes (Fig. 4). Eleven species indicated lakes and 23 indicated rivers (Table 3).

Nearly all sampling sites had local (HUC-12) catchment habitat composed of 93–98% native plant communities (Table 2), primarily eastern hemlock (*Tsugacanadensis*), northern white cedar *(Thuja occidentalis*), and white pine (*Pinusstrobus*), with occasional oaks (*Quercus* spp.) and maples (*Acer* spp.). Impervious surface was < 0.5% of all local catchment areas. Specific conductance ranged 40–100 µC/cm^2^ in lakes and 60–120 in streams; pH ranged 8.0–8.4 and 8.0–8.6 respectively, and dissolved oxygen ranged 7.2–8.1 ppm and 7.2–9.1 ppm. Most landscape variables exhibited minimal difference between sites.

## ﻿Discussion

Several unique species were collected during this study (Table 3). Specimens of *Cernotinapallida* (Banks) (Polycentropodidae), *Hydroptilafiskei* Blickle (Hydroptilidae), *Limnephilusfemoralis* Kirby and *L.thorus* Ross (Limnephilidae), and *Triaenodesperna* Ross (Leptoceridae) represent the only known collections of these species within Michigan. Both known Michigan endemic species, *Plectrocnemiasabulosa* (Leonard and Leonard) and *Setodestruncatus* Houghton, were also found during this study. The latter species is currently known worldwide only from the Pine (site 8) and Salmon Trout (17) rivers.

The known species richness of the Huron Mountains habitats represents > 50% of all 305 species found in Michigan (Houghton et al 2018; Houghton 2020) and > 30% of all ~ 550 species found in the Upper Midwest region of the United States (Rasmussen and Morse 2018; Houghton et al. 2022). The Huron Mountains habitats contained ~ 1.5 × as many caddisfly species (114) as the Black River Ranch of northern Lower Michigan, ~ 2.5 × that of Indiana Dunes National Lakeshore (64), and ~ 3.5 × that of Isle Royale National Park (46), other fairly undisturbed areas of Michigan and northern Indiana sampled with a rigorous effort (DeWalt and South 2015; DeWalt et al. 2016; Houghton 2016). The fauna of the Huron Mountains was more similar to those of the Black River Ranch and Isle Royale then it was to Indiana Dunes, with 8, 5, and 20 species found in the respective areas not found in the Huron Mountains. This result is not surprising given the similar latitude and terrestrial habitat of the Huron Mountains, Black River Ranch, and Isle Royale.

Habitat and water physicochemical data supported the undisturbed nature of Huron Mountains habitats, with high levels of intact native terrestrial habitat, low impervious surface, no historical or contemporary dams or human settlements, and low specific conductance values. Specific conductance is a general indicator of nutrient, sediment, and organic matter concentrations (Allan 2004). The values of HMC rivers were ~ 1/6 that of Michigan agricultural rivers (Castillo et al. 2000; Bernot et al. 2006; Arango et al. 2007; Houghton et al. 2011) and ~1/3 that of other undisturbed Michigan rivers (Houghton et al. 2018), suggesting very low anthropogenic seston enrichment. Yanoviak and McCafferty (1996) found similar low specific conductance values when they sampled the Pine River, Mountain Stream, and the Salmon Trout River ~ 27 years ago. The only stream site with < 93% intact native terrestrial habitat, Elm Creek (#15), had cattle grazing in its lower reaches > 100 years ago; such reaches were subsequently replanted with a wildflower meadow. While it is unlikely that any ecosystem in the contiguous 48 states of the US is in truly pristine condition, the habitats of the HMC probably represent some of the closest available to the original terrestrial and aquatic habitat conditions within the northcentral US (Flader 1983; Simpson et al. 1990) and are, thus, appropriate for determining reference conditions and differences in faunal assemblages between ecosystem types.

The separation of caddisfly species assemblages between lakes and streams despite their close geographic proximity supports the distinctness of lotic and lentic habitats. Of the 11 species that indicated lakes, over half were in the Leptoceridae, a family typically associated with lakes and slow-moving rivers (Wiggins 2004). Conversely, most of the species that indicated rivers were known rheophilic hydropsychids, glossosomatids, or rhyacophilids. Few previous studies (e.g., Kimura et al. 2006) have attempted to establish characteristic species assemblages or indicator species for lakes, and none has directly compared these assemblages to nearby rivers.

Despite the taxonomic differences between lakes and rivers, both total biomass and that of most individual FFGs were similar between the two habitat types. The higher biomass of filtering collectors in rivers was probably due to the flow velocity needed to inflate their capture nets (Wiggins 2004). The higher biomass of predators in lakes was greatly influenced by the predator *Oecetisinconspicua*, a highly abundant lentic species. Whereas riverine systems have had several models proposed that predict changes in FFG ecology based on stream size and other factors (Vannote et al. 1980; Thorp et al. 2006; Maasri et al. 2021), lake environments have received much less attention. Some previous studies have proposed that lakes, particularly eutrophic lakes, are primarily autochthonous (Francis et al. 2011; Galloway et al. 2014; Lau at el. 2014), while others have confirmed the importance of allochthonous carbon in supporting lentic food webs (Pace et al. 2004; Tanentzap et al. 2017). All such studies, however, focused on zooplankton instead of benthic insects. The high relative biomass of shredders (~ 50%) relative to scrapers (< 10%) in lakes of the Huron Mountains demonstrated the importance of coarse allochthonous input to lake food webs. While only caddisflies were sampled in this study, several other studies have demonstrated that trends in caddisfly FFG ecology usually reflect those of the overall insect assemblage (Mackay and Wiggins 1979; Dohet 2002; Houghton et al. 2011; Houghton et al. 2018; Morse et al. 2019a; Houghton 2021).

Due to the close proximity of sites in this study, it is likely that some specimens were sampled by a light trap of a different natural habitat. While this problem can never be completely eliminated, several studies suggest that the low vagility of caddisflies promotes minimal specimen ‘leakage’ between sampling sites (Sode and Wiberg-Larson 1993; Peterson et al. 1999; Sommerhäuser et al. 1999). Brakel et al. (2015), in particular, found a forest and meadow site of a Michigan stream separated by ~ 100 m had very little overlap in their adult caddisfly assemblages when sampled using ultraviolet lights. Further, the indicator species analysis (Dufrêne and Legendre 1997) employed in this study is negligibly influenced by occasional specimens. Thus, abundant riverine species such as *Cheumatopsychecampyla* Ross, *Hydropsychebetteni* Ross, or *H.morosa* Hagen constituted river indicator species, even though they occasionally were sampled at a lake.

Future research should include sampling caddisflies and other aquatic insects in remaining undisturbed habitats throughout the northcentral US and elsewhere. Observed differences of caddisflies between lakes and rivers would increase in value if also observed with other aquatic insect orders within other regions. Further sampling of lake habitats is particularly important so that models can be generated to predict changes in aquatic insect assemblages relative to specific lake variables.
